# Identification of Cichlid Fishes from Lake Malawi Using Computer Vision

**DOI:** 10.1371/journal.pone.0077686

**Published:** 2013-10-25

**Authors:** Deokjin Joo, Ye-seul Kwan, Jongwoo Song, Catarina Pinho, Jody Hey, Yong-Jin Won

**Affiliations:** 1 Department of Electrical Engineering and Computer Science, Seoul National University, Seoul, Korea; 2 Division of EcoScience, Ewha Womans University, Seoul, Korea; 3 Department of Statistics, Ewha Womans University, Seoul, Korea; 4 CIBIO, Centro de Investigação em Biodiversidade e Recursos Genéticos, Universidade do Porto, Vairão, Portugal; 5 Department of Biology, Temple University, Philadelphia, Pennsylvania, United States of America; CNRS, France

## Abstract

**Background:**

The explosively radiating evolution of cichlid fishes of Lake Malawi has yielded an amazing number of haplochromine species estimated as many as 500 to 800 with a surprising degree of diversity not only in color and stripe pattern but also in the shape of jaw and body among them. As these morphological diversities have been a central subject of adaptive speciation and taxonomic classification, such high diversity could serve as a foundation for automation of species identification of cichlids.

**Methodology/Principal Finding:**

Here we demonstrate a method for automatic classification of the Lake Malawi cichlids based on computer vision and geometric morphometrics. For this end we developed a pipeline that integrates multiple image processing tools to automatically extract informative features of color and stripe patterns from a large set of photographic images of wild cichlids. The extracted information was evaluated by statistical classifiers Support Vector Machine and Random Forests. Both classifiers performed better when body shape information was added to the feature of color and stripe. Besides the coloration and stripe pattern, body shape variables boosted the accuracy of classification by about 10%. The programs were able to classify 594 live cichlid individuals belonging to 12 different classes (species and sexes) with an average accuracy of 78%, contrasting to a mere 42% success rate by human eyes. The variables that contributed most to the accuracy were body height and the hue of the most frequent color.

**Conclusions:**

Computer vision showed a notable performance in extracting information from the color and stripe patterns of Lake Malawi cichlids although the information was not enough for errorless species identification. Our results indicate that there appears an unavoidable difficulty in automatic species identification of cichlid fishes, which may arise from short divergence times and gene flow between closely related species.

## Introduction

The African great lakes, including lakes Malawi, Tanganyika and Victoria, are grand reservoirs of species of fishes in the family Cichlidae. Within these lakes evolution has formed roughly 1450 to 1750 different cichlid species by rapid speciation [1]. Thus, these species flocks comprise a central subject of research in the field of evolution [2]–[4]. In Lake Malawi, numerous haplochromine cichlid species between 500 to 800 have been formed through adaptive radiation within the past million years from a few common ancestors from neighboring rivers with subsequent genomic contributions from river cichlids that currently survives outside the Lake Malawi catchment; these species have adapted to various habitats and diets with high diversity in morphology and coloration [5]–[8].

Coloration is the most salient variable character among congeneric Lake Malawi cichlid species and thus it has attracted many researchers' interests particularly in its role for sexual selection and thus for speciation [9]–[15]. Males and females are often strikingly sexually dichromatic; adult males generally exhibit bright and diverse colors, but females tend to have dull color. In addition to color information, the stripe patterns of cichlids, which involve various shapes and combinations of melanic bars, spots, bonds and divisions of different colors, constitute another layer of the phenotypic space of cichlids on which natural and sexual selection has probably been acting.

Our current approach to the examination of color components drew from two previous studies that directly examined the color features of cichlids [16], [17]. Both of these previous studies manually dealt with photographs for the extraction of color information without automation across photographs. The two studies provided no information about the variation within species, because comparisons between specimens were done at the species-level. [6]. Therefore it could be said that such diverse coloration has not yet been tested enough for its relation to taxonomical identities. The space of color variation at both intra- and inter-specific levels requires processing a large number of photographic images.

The principle and concept of such an high throughput image processing for the recognition of certain biological entities, which is described as a sort of ‘automated taxonomic or species identification’, was previously well established and practically led to the development of a series of systems for some taxonomic groups: insects, plants, spiders, planktons and so on (see [18] for a review). The ‘automated species identification’ has a primary motivation of reducing the burden of routine identification of vast numbers of specimens. This approach even further extended to the application to real organisms in the field, such as zooplankton [19]. Previous studies were mostly based on features associated with externally recognizable patterns of body and/or cell shape, such as wing, genitalia, cell and pollen, with limited cases where color was targeted by feature extraction [20]–[22]. Thus, we adopted the basic idea and work flow of the ‘automated species identification’ with appropriate adjustments toward our target objects, the colorfully diverse rock-dwelling cichlid fishes.

Other works such as [23]–[26] and [27] put emphasis on classifying fish species by computer aided discrimination. In [24], extracted features from speckle patterns were used to classify three species at fish markets. Similarly, Rova et al. [25] used stripe pattern to discriminate striped trumpeter and western butterfish in open waters through underwater video. Both shape and texture features were used in [26] to classify cod, whiting and haddock. However, the previous works have applied computer vision (CV) techniques on species that look very different and is not applicable in close species discrimination which look very similar even to naked human eye.

To tackle the problem of classifying closely related species with the aid of computer discrimination, we developed an automatic classification method incorporating CV for the analyses of multiple features of coloration and stripe patterns of cichlid fishes of Lake Malawi in combination with geometric morphometric (GM) body shape information, expecting that this combination would maximize the resolving power of automatic classification. We assessed the performance of various options and parameter sets at each step of the image processing pipeline to determine the best conditions for the analysis of photographic data of cichlids. Subsequently, multiple features of coloration (*n* = 42) and stripe (*n* = 6) patterns of cichlid digital images and geometric landmarks (*n* = 17) of fish body shape were chosen for their appropriateness for the current purpose and evaluated their contribution to the accuracy of species and sex identification. Finally, we discuss how we can extend the usage of the current automated feature extractor and species identification for future study of evolution and speciation of cichlids, which will have implications for other animal studies.

## Materials and Methods

### Ethics Statement

The Institutional Animal Care and Use Committee, which oversees animal experimentation at Ewha University, was not established when this study was conducted. However, we treated our study subject, haplochromine cichlids, in strict accordance with the recommendations of the Animal Behaviour Society [28]. All the haplochromine cichlids examined in this study are not listed as endangered species and, in fact, are some of the most abundant shallow water fishes in Lake Malawi, Africa. All the fish examined were returned to the lake alive.

Our field sampling of cichlid fishes in the Lake Malawi was properly permitted by the Malawi Government. For this evidence we provide a copy of special licence granted to us by the Senior Parks and Wildlife Officer of Lake Malawi National Park on May 30, 2008. According to this, we were allowed to collect approximately 1500 individual fishes of rock-dwelling cichlids locally called as Mbuna and to take fin clips from them for genetics work later in laboratory. The Mbuna cichlids are not endangered so there are no national (in Malawi) or international (in Korea or Portugal) prohibitions to sample them for scientific research.

At the field, we took care to anesthetize the fish before clipping fins for genetic study in the future. Our experimental protocol at the field was as follows; prior to photographing, the fishes were anaesthetized in a basin with natural clove oil. Besides facilitating the handling of the fish, this step ensured that, upon relaxation of the animal, colors were fully expressed, thereby reducing the noise in color pattern variation associated to different emotional states. Small portions of the dorsal and caudal fins were clipped for genetic analysis in the laboratory later. Finally, the fishes were transferred to a recovery basin protected from any perturbation triggered by other wild animals and human activities, then safely recovered there. All the fishes were recovered within less than 20 minutes in the recovery basin, and then freely swam away to the lake.

Regarding the survey research with 10 volunteers, all non-experts in taxonomy of cichlids, we carried out the survey at Ewha Womans University in August and October, 2011, separately twice with two groups, each 5 persons. The purpose of this survey was to assess how accurately non-experts could identify 12 classes of images of cichlid fishes compared to the computer vision method that we developed for automated species identification. Our manuscript was mainly allocated to the explanations about a new method for species identification by computer aid. Therefore we were interested in whether the performance of the new method was acceptably high compared to the performance of non-experts in taxonomy. We properly explained the purpose to the 10 participants before the experiment and obtained their verbal consents for participating in our survey. Because the purpose of this survey was to obtain rates of misclassification of color-printed images from the ten volunteers and this whole process did not involve any violation of human rights and privacy, we did not obtained an official approval from the Institutional Review Board (IRB) at Ewha. Although the student volunteers spent approximately 90 minutes classifying cichlids, they did not suffer any physical or emotional pain. In addition, any private information from them was not obtained for any purpose of analysis and for publication after the survey. Additionally when we performed this survey in 2011, taking a review process of such survey that did not involve biomaterials from humans was not obligated in both Ewha and Korea.

The act on bioethics and safety has been revised on February 2, 2013 in Korea. Since that time, the legal enforcement of the bioethics and safety act began to operate in every institution through the organization of IRB. The IRB at Ewha Womans University was established on March 19, 2007, with enactment of 12 provisions of regulations on biomedical research activity involving humans. The regulations have been imposed to research activities involving invasive effects on humans. The 6th provision of the regulation describes seven different committees reviewing the following experiments that are relevant to the safety of human and ethics: generation of human embryos, research of human embryos, somatic human embryonic cloning, genetic testing of human, genetic research with biological materials originated from human, DNA bank of human genetic resources, and gene therapy of human disease. Although our study includes human survey on ten volunteers' capability in sorting printed images of wild cichlid fishes, it did not involve any concern that the seven committees routinely review.

### Study Species

**Figure 1:**
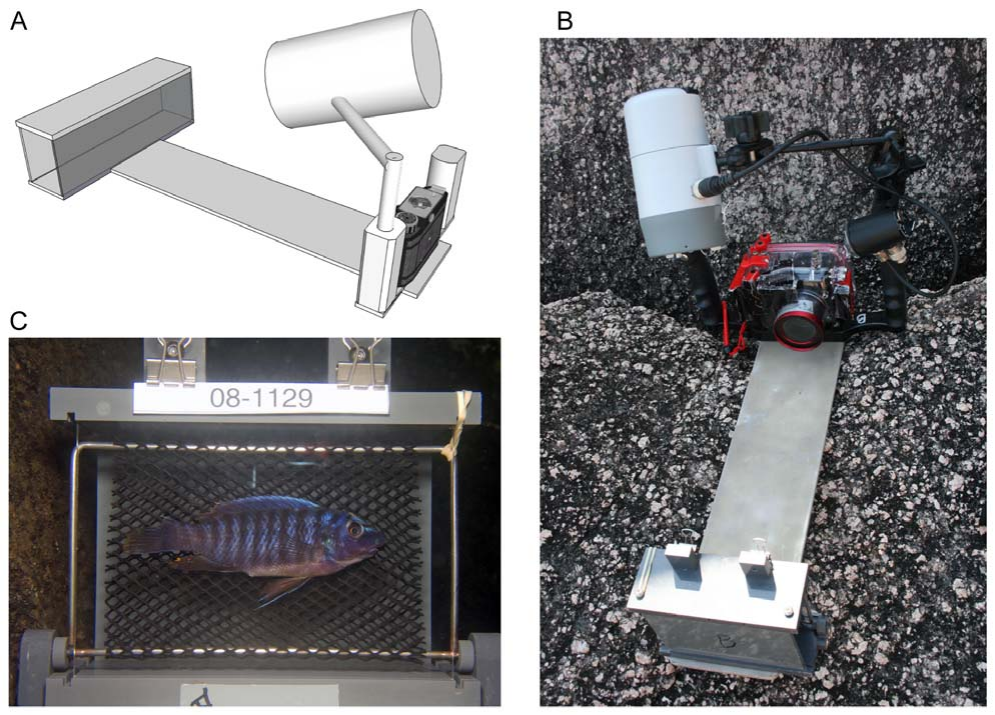
Apparatus for photographing anaesthetized fish underwater in repeatable circumstances and a raw image of photograph. (A) A schematic diagram and (B) a photograph of the camera apparatus. (C) This is an example of photograph taken from *Labeotropheus fuelleborni* (male). The number 08–1129 shown at the top of the photo is the identification number of this sample, and along with this label, useful information such as its species identification and the place where it was sampled has been recorded in spreadsheets. The 30% gray tape is shown at the bottom. The tape may be used as a reference in color correction, but it was not exploited in this study. Note that tissue samples (the upper portion of the caudal fin and the posterior portion of the dorsal) were removed before the photograph was taken.

We photographed live rock-dwelling cichlid fish in Lake Malawi from May 25 to June 20, 2008. Photographs and other ecological information were collected for every fish sampled. Fish were collected in two 5 m wide transects on the western shore of Domwe Island in southern Lake Malawi. Each transect extended from the shoreline to the bottom at approximately 20 meters of depth. Fish swimming within transects were caught by sweep netting in rough proportion to their actual occurrence and without regard to sex, species or feeding and territorial behaviors. We used monofilament nets about 3 by 3 m in size and SCUBA diving. We chose some species for further analyses based on their abundance and easiness to sample. In order to take photographs underwater controlling unwanted noise in pose and illumination, we developed a specialized camera platform that was intended to provide the same fixed distance from the camera lens to fish, uniform exposure and the same exact background (Fig. 1A & B). A large underwater strobe flash with the exposure controlled by the camera was used to ensure common and uniform light intensity regardless of cloud cover and water depth. To ensure accurate record keeping, we had serial numbers printed in waterproof ink separately and used them as identification numbers for each fish by attaching them to the upper part of the transparent sample box where fishes were gently held against polycarbonate by flexible gray mesh (Fig. 1C). The camera was a five-megapixel Olympus model in an underwater case. Prior to photographing, the fishes were anaesthetized in a basin with clove oil. Besides facilitating the handling of the fish, this step ensured that, upon relaxation of the animal, colors were fully expressed, thereby reducing the noise in color pattern variation associated to different emotional states. Finally, the fishes were transferred to a recovery basin and then returned to the site where they were collected. The identification of species and sexes were performed with the aid of photographs [6] and the help of two highly experienced local divers, Richard Zatha and James Maluza.

### Approach of Computer Vision to Cichlid Images

**Figure 2:**
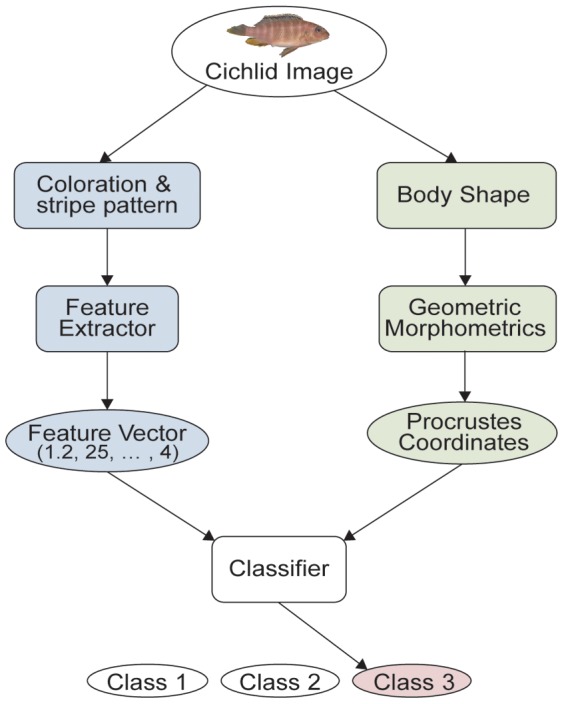
Overall flow of the pipeline. Cichlid images are fed into the feature extractor to be converted into vectors and are also subjected to the analysis of landmark-based geometric morphobetrics. Each vector is a numerical “summary” of each respective image and each Procrustes coordinates are numerical shape variables obtained from the landmarks of each image. With the summary and Procrustes coordinates, the classifiers, the Support Vector Machine (SVM) and Random Forests (RF) in this study, try to determine to which class the input vector belongs.

We designed a pipeline of the present study as shown in Figure 2 and assessed each step of it in relation to optimal conditions for its final performance in classification of photographic images of cichlids into their ascertained species and sex identity. The feature of coloration of fish images was captured by Red-Green-Blue (RGB) and Hue-Saturation-Value (HSV) color models and that of stripe patterns was captured by some proxies for the complexity of images.

In computer vision (CV) methods, a “feature” is a value or values extracted from a digital image to provide a summary in numerical form. For example, an average RGB value of the image, image dimension (width and height) and dimension ratio (width/height) could be used to summarize an image. The advantage of this approach lies in its simplicity and speed [29]. A feature extractor was programmed in Python programming language. The OpenCV library (http://opencv.willowgarage.com/wiki/CiteOpenCV) was employed to deal with image processing; this library can be thought as a programmable version of Photoshop. Some of the basic capabilities of OpenCV include counting colors, finding edges, converting color images to grayscale images and detecting straight lines.

A statistical classifier examines and maps sets of the features extracted and numerically summarized by CV to classes. For classifiers, artificial neural networks (ANNs) have been widely employed [30]. By mapping outputs to classes, the ANN can perform classification. Support vector machines (SVMs) [31] are close relatives of ANNs in terms of input and output behavior. They take many inputs and perform classifications. SVM tries to find the decision boundary that has the maximal margin. A margin is the distance between the decision boundary and the closest observation. In fact, some configurations of SVMs are mathematically equivalent to some configurations of neural networks. Specifically, Collobert et al. [32] showed that Perceptrons (two-layer neural networks) are equivalent to linear SVMs. However, SVMs and ANNs have different learning methods, and SVMs have a sounder mathematical base. ANNs adjust the weights of each neuron, while SVMs find hyperplanes to learn. In this study we chose and compared two different classification methods, support vector machines (SVM) and random forests (RF), because they have performed well in many applications. The high usability and flexibility of SVM has resulted in a list of growing number of successful applications for various tasks of prediction and classification of biological units: DNA barcodes [33], microRNAs [34], fluorescent microscopic images [35] and even the classification of remote-sensing images [36]. Random forests (RF) is a bagging tree method that uses many bootstrap samples and trees [37]. It uses random sample of variables instead of all variables for each tree to reduce the correlation among the trees. Recently RF is widely used for image classification [38], ecology [39], and medical genetics [40], [41]. Unlike SVMs, RF can identify important variables for classification. This information can be used for further study.

For the SVM classifier we used LIBSVM [42], which is widely used and has many scripts for automating common tasks, such as selecting a *training set* (see following section). The number next to the feature vector in Figure 2 is a one-dimensional list of numbers obtained by the feature extractor as a numerical summary of the source image.

Preparation of the SVM and RF involves a training phase, during which the classifiers are changed as a function of the learning material. To optimize the process and adjust the parameters, several questions must be answered: Which features should be extracted? Which features provide more information for the classifiers? Is any image of a cichlid acceptable? These questions are addressed in the following sections. The programs and script used in this study are available for download from http://code.google.com/p/ghoti/ or by contacting Joo (djin.joo@gmail.com).

### Image Preprocessing

Before using the feature extractor, input images were preprocessed to improve the quality of extracted features.

#### Image selection

**Figure 3:**
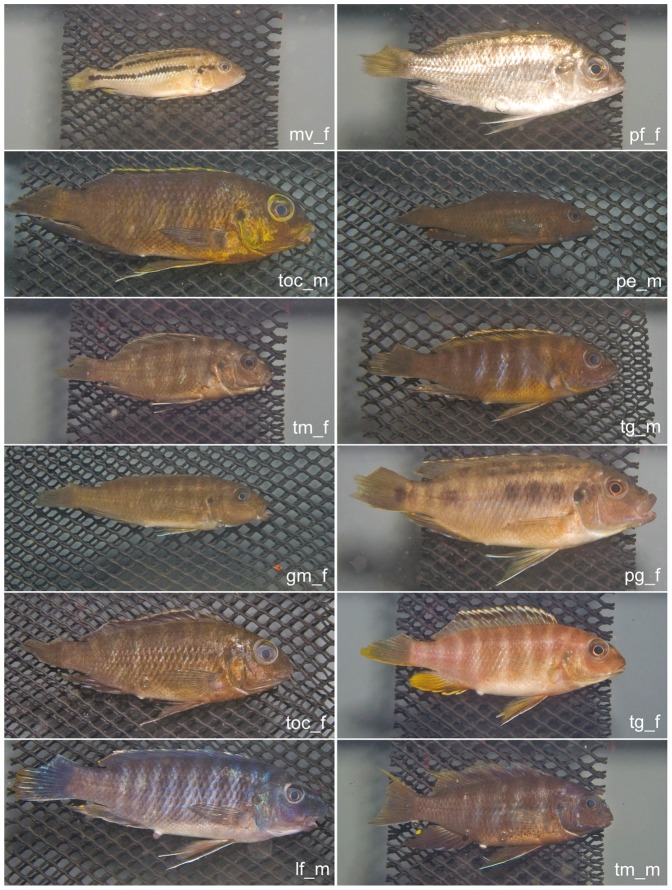
Representatives of each of 12 different classes of cichlids. These rock-dwelling cichlid species had relatively more photo samples per group (>10) and were chosen for the analyses of feature extraction and classification as illustrated in Figure 4. The name tags in the lower right portion of each photograph are combinations of the abbreviation of species names (Table 1), followed by “m” for male and “f” for female.

To check for errors in the initial identification of species and sex at the field, the photographic images were grouped and separated by their classes for manual examination. Due to the practical nature of the sample collection and photographing live fishes, photographic images that were deemed likely to be misidentified were excluded from the study rather than reclassified. Juvenile samples were removed because they did not exhibit the traits of fully mature cichlids. Apart from these factors, blurred images had to be excluded because they were not suitable for extracting features that require sharp color transitions, such as edge detection and line detection. In this study we examined 594 individual fishes belonging to 9 species of 12 classes that had “enough” samples, that is, no less than 10 samples per class (Table 1 and Fig. 3). As mentioned above, we treated male and female individuals as different classes due to their sexual dimorphism for coloration [6], although taxonomically they belong to the same species. Hereafter all the names of the classes were abbreviated and followed by identify of sex for brevity; *Tropheops* sp. “orange chest” female and male were represented as toc_f and toc_m, respectively. The “_f” and “_m” stands for female and male, respectively.

**Table 1:** Summary of species name abbreviations and number of samples examined by the SVM and RF classifiers.

Abbrev.	Name	#Female	#Male
gm	*Genyochromis mento*	11	-
lf	*Labeotropheus fuelleborni*	-	12
mv	*Melanochromis vermivorus*	11	-
pe	*Pseudotropheus elongatus*	-	30
pf	*Protomelas fenestratus*	11	-
pg	*Petrotilapia genalutea*	26	-
tg	*Tropheops gracilior*	188	90
tm	*Tropheops microstoma*	20	14
toc	*Tropheops* “orange chest”	113	68

#Female and #Male columns show the composition of the species in the vector pool. The species and sexes are arranged in alphabetical order.

#### Color balancing/enhancing

The photographs were taken with 30% neutral gray as the reference color, as shown in Figure 1C, enabling the use of a color balancing technique. However, this processing requires a great deal of human effort and introduces another human uncertainty into the process; depending on the selection of the pixel, the image is altered considerably. To examine the effect of automatic color correction images were processed using ImageMagick's [43] normalize command: “convert -normalize input.jpg output.jpg”. However, this treatment had a negative effect on the final performance of the SVM classifier, degrading accuracy by about 10% due to image-wise application of color correction, resulting in inconsistency through images. In the final study no color correction was applied.

#### Removing background from images

**Figure 4:**
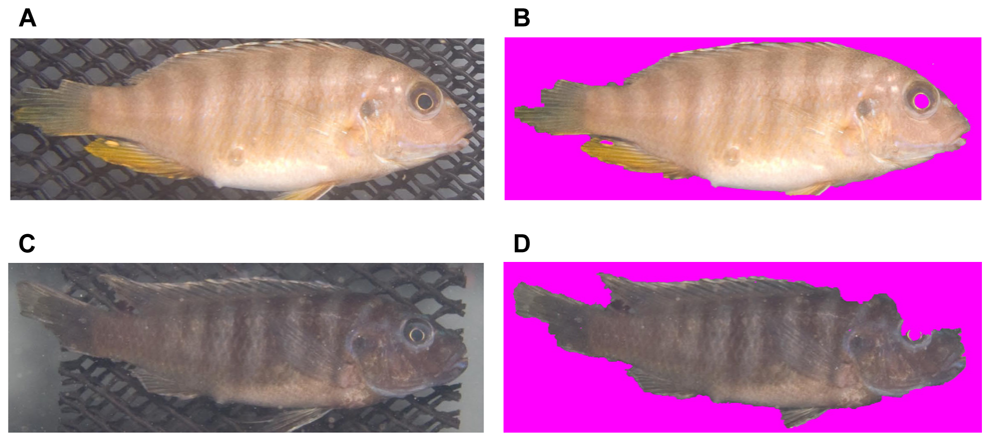
Grabcut results. Images in the left column are the inputs to the Grabcut algorithm, and the images on the right column are their results. The detected background was removed and replaced with magenta, which was ignored by the feature extractor. The Grabcut algorithm works more effectively than the conventional magnetic lasso or magic wand tool. However, no algorithms are perfect, and they may yield poor results, similar to (D).

To isolate the pixels of a sample from the surrounding image the Grabcut [44] algorithm was used and the implementation can be found at [45]. The Grabcut algorithm works by considering both brightness changes and color similarity, lending better results than many other classic background removal tools. Figure 4 shows examples of results of background removal. Bright-colored cichlids which can be easily distinguished from the background, yield good results while dark-colored cichlids are prone to yield poor results, like the example shown in Figure 4D. However, even if the image of the fish was imperfectly isolated from the background, we still used the image because the majority of the cichlid's body was preserved, and the features to be extracted were chosen to be less sensitive to the geometric details.

### Feature Extractor

The feature extractor was programmed in Python programming language using the OpenCV library. The feature extractor takes in images and extracts a numerical summary of each image in vector form (Fig. 2). The extracted features were largely inspired by Rowley et al.'s [29] work. Up to 48 features were extracted by the extractor.

#### Color information

The digital image is first quantized (i.e. similar to the *posterize* or o*ptimum palette generation* commands in image editors) into 7 colors, which can best represent the real colored image, excluding the background color, magenta. Each color is represented in RGB and HSV, yielding 7×(3+3) = 42 features.

#### Color ratio

Another color related feature is the ratio of the number of pixels which are in the most frequent color and the second most frequent color. For example, if 600 pixels are brown and 500 pixels are blue, and no other colors have more than 500 pixels, then the extracted feature is 1.2 ( = 600/500). This ratio is extracted in the hope that it would provide information about the cichlid's stripe pattern.

#### Entropy

Entropy can be roughly explained as the complexity of an image. The higher the entropy, the more complex is the image. The entropy is extracted in the hope that it would provide information about the complexity of the cichlid's stripe pattern. The input image is first converted to an 8-bit grayscale version, and the entropy value is calculated by equation 1: 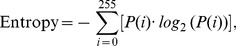
(1)where 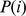 is the fraction of pixels with intensity *i*.

#### Edge pixel count

**Figure 5:**
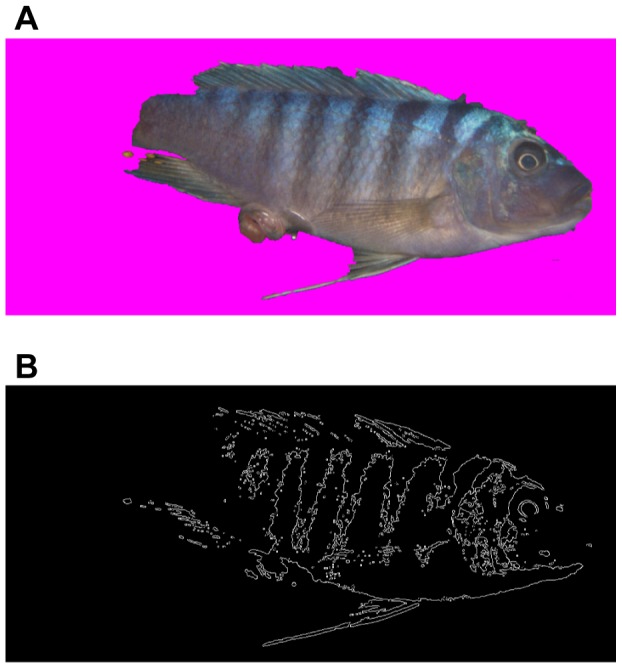
An example input image and its resulting edge detection. (A) A photographic image before edge detection and (B) after a canny edgy filtering. Places where brightness changes suddenly are marked white.

In image processing, pixels where the brightness is radically different from those of nearby pixels may be detected and called edges. Figure 5 shows an instance of edge detection run on a cichlid image. Edge detection is employed as another method of describing the complexity of the stripe pattern; the number of edge pixels is recorded. In this implementation of the extractor, a canny edge filter is used. However, larger species tend to have longer regions of edges. To compensate for this, the value defined by equation 2 is used instead: 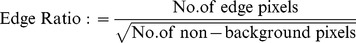
(2)


#### Line features

As a more direct method of detecting the stripe pattern, we detect straight lines from the edge detected image, using the OpenCV implementation of the probabilistic Hough transform, with an angular granularity of 45 degrees [46]. Hence, three features are extracted: the number of horizontal lines, vertical lines and diagonal lines. The Hough transform extracts straight lines from man-made objects quite well, while it has poor performance on natural objects. Cichlids are no exception, and the detection result is very poor. Nonetheless, line features do contribute about 1 to 2% accuracy to the classification result.

#### Feature extractor parameters

The feature extractor has many internal parameters for each feature. It would be unnecessarily verbose to describe here; thus, we have provided the extractor available for download at http://code.google.com/p/ghoti/.

### Landmark-based Geometric Morphometrics (GM)

Seventeen landmarks outlining body shape and position of fins and organs were designed as similar as previous studies with cichlid fishes (Fig. S1). The *x*, *y* coordinates (*n* = 34) of 17 landmarks were digitized from the photo of each individual using TPSDIG32 (ver 2.12) [47]. Generalized Procrustes analysis [48] which removes non-shape variations by rescaling each fish to unit size and rotating images so that each corresponding landmarked positions are aligned as closely as possible was applied to the coordinate data to extract shape variation using MorphoJ (ver 1.03a) [49]. For SVM and RF classification, the resultant *x*, *y* Procrustes coordinates were appended to the numerical matrix of 48 features extracted as above by the feature extractor.

### Classifiers

#### Vector pool generation for SVM

A preprocessor was programmed to enable vector selection and feature selection. For example, one might want to remove all females and use only males because males seem to have more distinct features from species to species. Also, in some experiments it may be desirable to disable the use of color features to examine the usefulness of non-color features. Another important preprocessor is the LIBSVM tool svm-scale which examines the vectors and scales all the attributes to intervals [0,1] or [−1,1]. Without this scaling, some large valued attributes may dominate over small valued attributes, rendering them insignificant. The raw vector from the extractor is processed by the custom preprocessor, and it is then scaled by svm-scale.

#### Training and classification parameters of SVM

Like the feature extractor, LIBSVM requires many parameters. For actual parameters used in the experiments, refer to the scripts at http://code.google.com/p/ghoti/.

#### Cross-validation of SVM

We partitioned the 594 vectors (number of specimens, Table 1) randomly with 80% training set and 20% test set and fit models in a training set and computed the misclassification rates in a test set. Because the two sets are disjointed, the test set can act as a new “unknown” vector for the SVM to predict. We repeated this procedure 100 times, leading to 11,800 predictions in total. For automated iteration of training and classifications, we made scripts that executed programs in the LIBSVM tool set. The results of the classification can later be examined to construct a *confusion matrix*. Confusion matrices will be discussed in the Results section.

### Random Forests (RF)

The dataset had 594 observations and 82 variables of which 34 came out of *x*, *y* coordinates of 17 geometric landmarks. The response variable was the 12 different classes. We tried to find the optimal models using 10 fold CV. We found that RF with the number of randomly selected variables  = 14 and the number of trees  = 500 gives the best result. We performed a cross-validation of RF in the same way described above in SVM.

### Survey Research

As an alternative way to evaluate the performance of computer vision in fish classification, we surveyed ten volunteering undergraduate students about how accurately they could discriminate the 12 different classes of cichlids using only the naked human eye. They were separately consulted about species and sex identification of the images. None of students had any formal training in fish classification before this survey. They were divided into two groups and then tested with different type of image sets, respectively: the original photo images with background (e.g. Fig. 4A & C) and the background-subtracted ones (e.g. Fig. 4B & D). We printed in color all the 594 cichlids with the image size being 12 by 8 cm size and provided each participant with randomly selected but presorted 12 classes of fish images of 421 individuals, about 70 percent of the 594 individuals, so that he/she had one hour of learning time to sufficiently perceive and compare various features of each fish groups by him- or herself. Then, the participant was asked to classify the rest of the fish samples (173 individuals) for 40 minutes. The students were allowed to refer back to the 12 presorted classes continuously while trying to sort the images one-by-one. We counted and summarized the number of accurate classification and misclassification out of the 173 tests per person.

## Results

### Classification

#### Performance of the extractor

The extractor was run on the 594 individual images of cichlids, each about 1100×400 pixels, on a computer with an Intel Core i5 CPU running at 2.40 GHz and 2 GB RAM. The task took 13 minutes, 15 seconds; thus, the average time to process 1 image was about 1.2 seconds.

#### Feature selection

**Figure 6:**
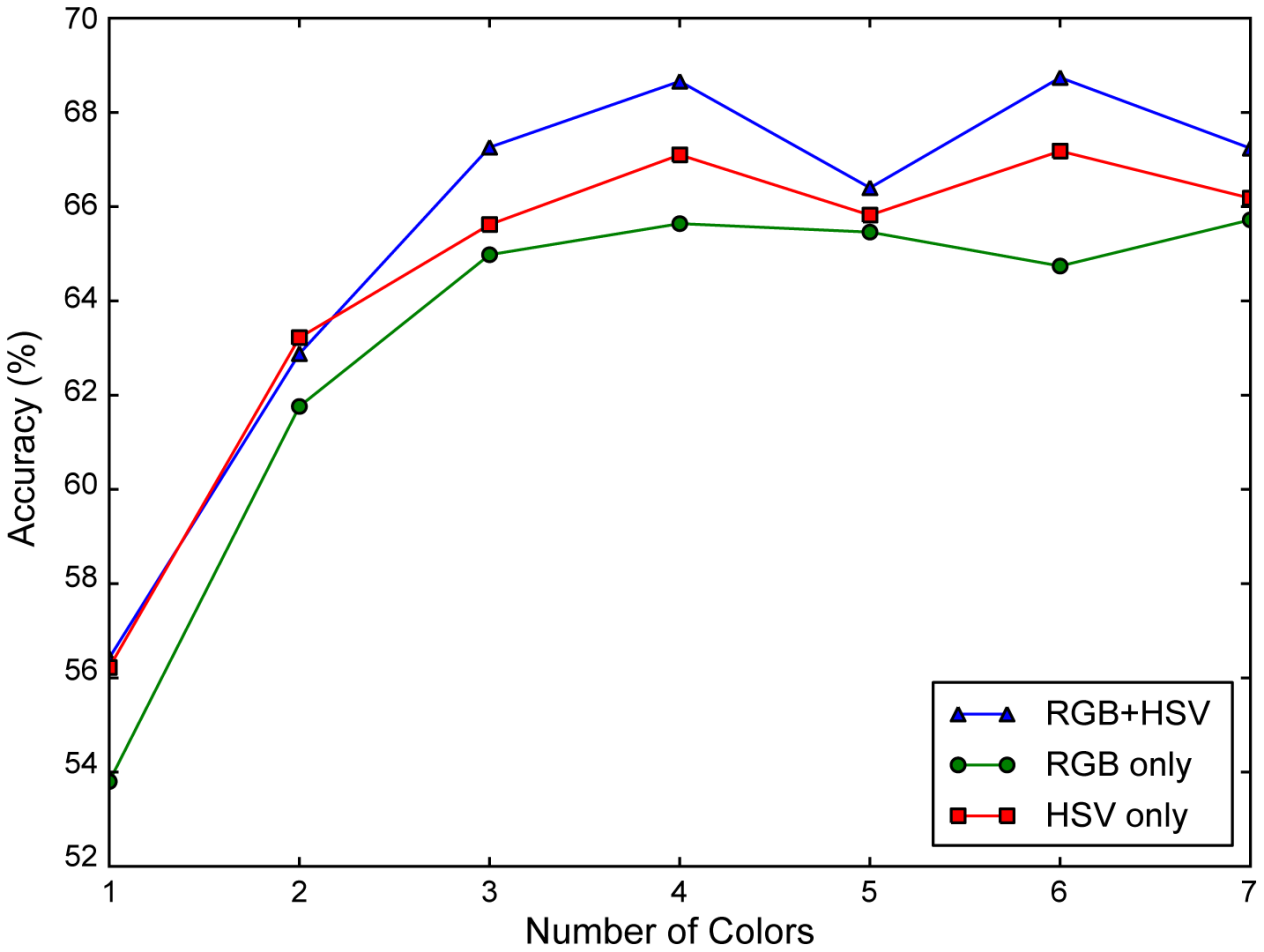
Number of colors used for training vs. classifier accuracy. If the number of colors used is 4, this means only the 4 most frequent colors from 7 extracted colors are used for training the classifier. No other features besides color were used to obtain this plot.

Figure 6 shows the SVM classifier accuracy vs. the number of colors used for training. No other features besides the colors were used to obtain this plot. The *x*-axis indicates the number of colors used for training the classifier. When the number of colors is 1, only the most frequent color is used, and when it is 2, the first and the second most frequent colors are used, etc. As seen in the figure, colors alone are a useful set of features, contributing more than 50% of the accuracy of the classification, even if only the most frequent color is used. Using more color does not necessarily lead to better classification. In addition, rather than RGB or HSV alone, using RGB *and* HSV values simultaneously can improve the overall performance of the classifier. It seems to be due to the fact that while HSV values can be calculated from RGB values, their relation is non-linear; hence, providing both RGB and HSV values delivers extra information to the classifier, which works linearly.

Similarly, providing more features does not necessarily improve overall performance. Therefore, it is necessary to explore the feature combinations and identify features that confuse the classifier and discard them. However, it is infeasible to try every combination of features because we have 2^48^ possible combinations. To accommodate this problem, a simple heuristic method suggested by Chen and Lin [50] was used. An F-score was calculated for each feature, and only the features with F-scores greater than or equal to some user determined threshold value were selected, where the F-score of feature *i* is defined by equation 3: 
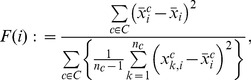
(3)where *C* is the set of the classes, 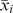 is the average of the feature *i* in all classes, 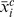 is the average of the feature *i* within a class *C*, *n_c_* is the number of vectors that belong to class *C* and 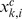 is the value of feature *i* in vector number *k* of class *C*. Simply put, the denominator is the sum of the variance of each class. All features from the 3 most frequent colors had F-scores greater than 1, while most of the least frequent 3 colors had F-scores less than 0.5; most of the scores were about 0.1. Generally, features with higher F-scores are more likely to be more discriminative.

Table 2 presents F-scores of non-color features. We used features with F-scores greater than 0.7; hence, the color ratio and diagonal line features were excluded. Classification with feature selection yielded an accuracy of 68 to 69% by the SVM classification, while without selection, it yielded an accuracy of 67%. It is worth noting that without any color features, these features can achieve an accuracy of 48%, and the accuracy with only color features is 66%. Note that these accuracy ratings represent average accuracies acquired from 100 repetitions of randomized tests, and they suffer from Monte Carlo variation.

**Table 2:** The F-scores of features.

Feature	F-score
Color ratio	0.42
Entropy	0.98
Edge count	1.08
Horizontal line	0.93
Diagonal line	0.46
Vertical line	1.15

F-scores for non-color features. Features with higher F-scores are more likely to provide useful information to the SVM classifier.

#### Color information

It is worth noting that the previous study of cichlids [17] focused on single average color of limited areas of body. However, in this study, rather than simple averaging, a color quantization method has been used to cluster colors in groups. Figure 6 suggests that groups of color can deliver better performance than one average color. This suggests that multivariate analysis should make use of more information than was used in previous works.

**Figure 7:**
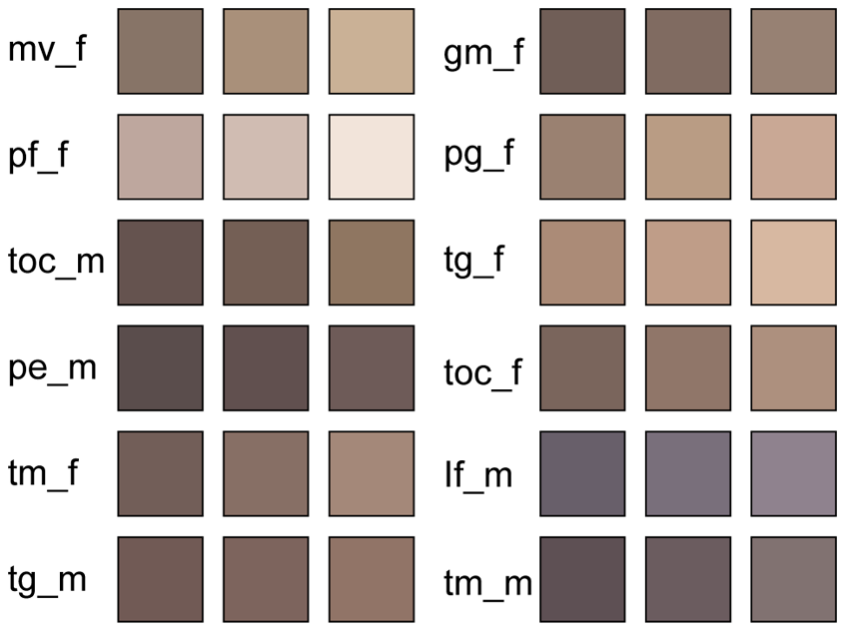
Palettes of the three most frequent color components among the vectors extracted by the feature extractor from the 12 different cichlid classes. Each class is labeled according to Table 1 and followed by a tag for male (_m) and female (_f), respectively.

**Figure 8:**
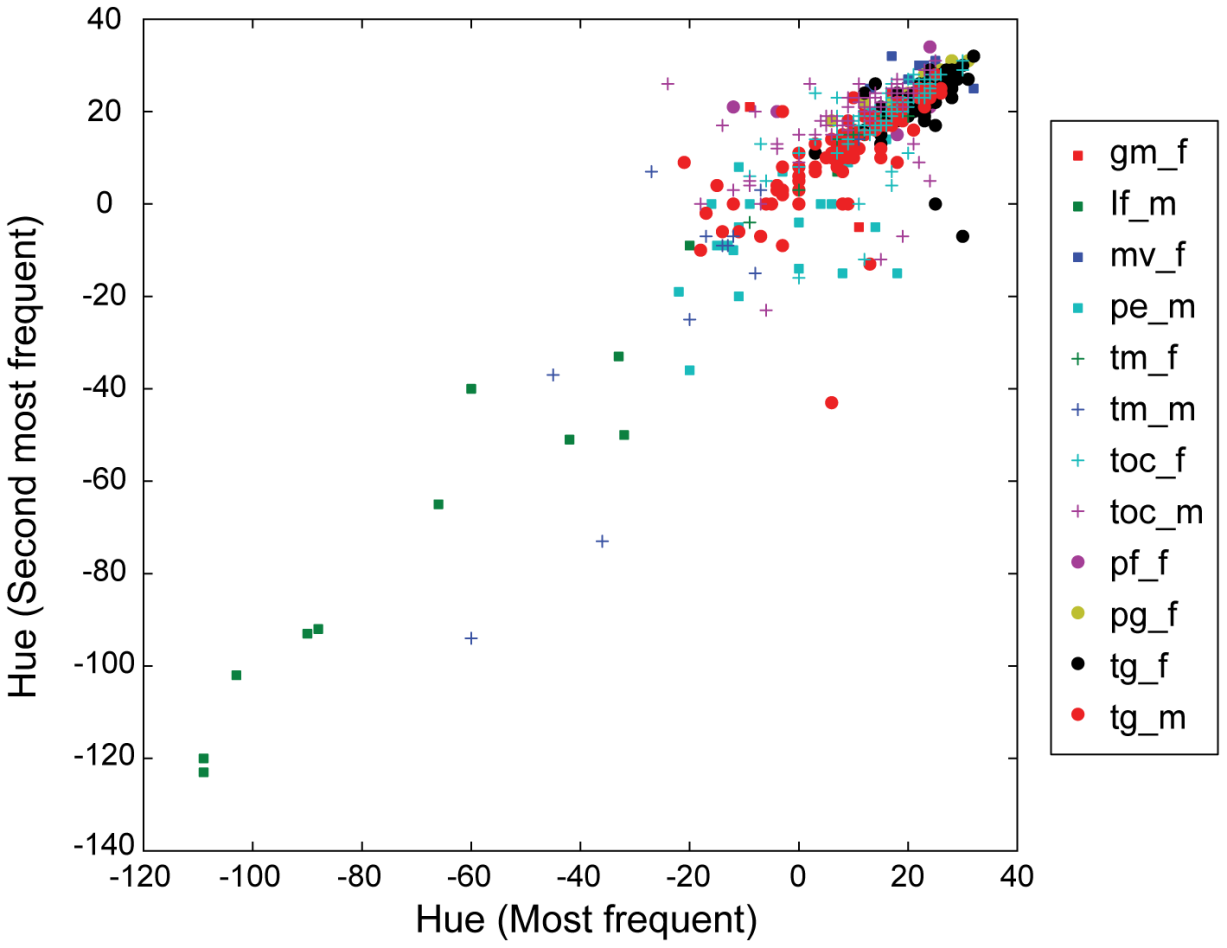
Plot of the two most frequent colors among species. The plot conforms to Deutsch's observation [26] which states that all significant variation in male cichlid color occurs within species, rather than between species.

To visualize the high efficiency of using some proportionally frequent color components, we averaged the three most frequent colors and condensed them into a palette (Fig. 7). When only one color is given, all colors appear brown tints that are quite similar (results not shown). However, given the three most frequent colors, we get the impression that we can distinguish the cichlids fairly well. All significant variation in male cichlid color occurs within species, rather than between the species (Fig. 8).

#### The confusion matrix

One hundred results of randomized repetitions of SVM and RF classification on different types of data (colors, stripe and GM) provided summary statistics of both mean and standard deviation (s.d.) of the accuracy (Table 3). The most accurate classification was obtained by SVM classification (average  = 77.6%; s.d.  = 3.4%) when the body shape information based on landmark-based GM was combined to the vectors of 48 different features of colors and stripe patterns. Random forests (RF) also yielded a very similar result with the same data (Table 3). SVM slightly gave better result than RF. When only one type of data was used, the success rate ranged from 64% to 68% (Table 3). SVM on color and stripe yielded an accuracy of 78.2% when only female samples were used, and using only males yielded 77.5% (data not shown). This means that separating the sexes yielded more accuracy than using both by presumably lowering the total number of classes, which had an accuracy of 68 to 69%.

**Table 3:** Accuracy of classification based on different information and methods.

	Color and stripe	Body shape	All combined
SVM	68.1 (3.6)	66.7 (3.7)	77.6 (3.4)
RF	64.8 (4.0)	64.1 (4.1)	74.5 (3.9)

Statistics in percentage (%) of mean and standard deviation of 100 results of 100 repetitions of randomized tests. The numbers in parenthesis are standard deviation.

**Figure 9:**
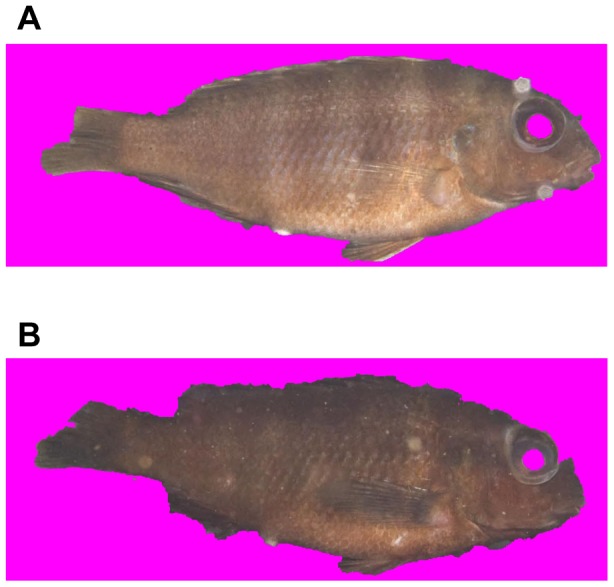
An example of the most frequently confused images. (A) A sample image of tm_f and (B) toc_f.

Table 4 presents a confusion matrix acquired from the repetition of the randomized subsampling cross-validation tests (*n* = 11,800). The table has been made by counting the predictions by SVM classifier. For example, the number 137 at column tg_m row toc_m represents that the classifier mistook tg_m for toc_m 137 times during 11,800 classifications. Note that these accuracy ratings represent average accuracies acquired from 100 repetitions of randomized tests, and they suffer from Monte Carlo variation. Notably, tm_f showed the lowest accuracy and were instead frequently classified as toc_m & _f. Figure 9 shows a sample image of tm_f and toc_f. Because the species are very similar, the extracted vectors are inseparable in the vector space. For such case, the classifier places more weight on toc_m & _f because there are more toc_ms & _fs than tm_fs. Among the frequently misclassified classes, tm_f always recorded the first one and other classes such as tm_m and toc_m followed it regardless of the data types and the classification methods (Table 4, 5, and S1).

**Table 4:** Confusion matrix of SVM on coloration and stripe vectors without GM information.

Predicted	Actual											
	gm_f	lf_m	mv_f	pe_m	pf_f	pg_f	tg_f	tg_m	tm_f	tm_m	toc_f	toc_m
gm_f	128	0	0	32	0	0	0	46	0	0	0	0
lf_m	0	210	0	0	0	0	0	0	0	0	0	0
mv_f	0	0	154	0	0	0	0	0	0	0	0	0
pe_m	42	0	8	443	0	0	13	56	0	0	0	1
pf_f	0	0	13	0	205	1	0	0	17	0	0	12
pg_f	0	0	3	0	0	514	0	10	0	0	0	14
tg_f	0	0	7	86	0	0	3058	605	41	0	357	113
tg_m	21	0	18	47	0	6	444	860	30	0	97	161
tm_f	0	0	0	0	14	0	5	1	72	60	44	64
tm_m	0	0	0	0	1	0	0	0	43	72	8	47
toc_f	0	0	0	3	6	13	185	76	83	30	1535	317
toc_m	0	0	0	0	3	2	39	137	87	94	253	633
Sum	191	210	203	611	229	536	3744	1791	373	256	2294	1362
Accuracy(%)	67.02	100	75.86	72.5	89.52	95.9	81.68	48.02	19.3	28.13	66.91	46.48

By 100 repetitions of randomized experiments, 11,800 predictions were made. An average accuracy of the result is 66.49%. The species and sexes are arranged in alphabetical order as in Table 1.

**Table 5:** Confusion matrix of SVM on the combined vectors of coloration, stripe and GM information.

Predicted	Actual											
	gm_f	lf_m	mv_f	pe_m	pf_f	pg_f	tg_f	tg_m	tm_f	tm_m	toc_f	toc_m
gm_f	134	0	1	50	3	0	0	24	0	0	0	0
lf_m	0	211	0	0	0	0	0	0	0	19	0	0
mv_f	14	0	202	0	0	0	0	0	0	0	0	0
pe_m	42	0	0	387	0	0	0	61	0	0	0	11
pf_f	0	0	3	0	212	0	0	1	0	0	0	0
pg_f	0	0	0	0	0	480	0	0	0	0	0	20
tg_f	0	0	31	24	0	4	3455	232	19	0	215	17
tg_m	19	0	0	98	0	27	166	1193	1	11	120	85
tm_f	0	19	0	0	0	0	5	21	108	65	43	109
tm_m	0	0	0	0	0	0	0	0	30	155	1	3
toc_f	0	0	0	2	1	8	161	134	162	26	1654	349
toc_m	0	3	0	2	3	2	14	113	63	34	186	732
Sum	209	233	237	563	219	521	3801	1779	383	310	2219	1326
Accuracy(%)	64.11	90.56	85.23	68.74	96.8	92.13	90.9	67.06	28.2	50	74.54	55.2

By 100 repetitions of randomized experiments, 11,800 predictions were made. An average accuracy of the result is 75.62%.

**Figure 10:**
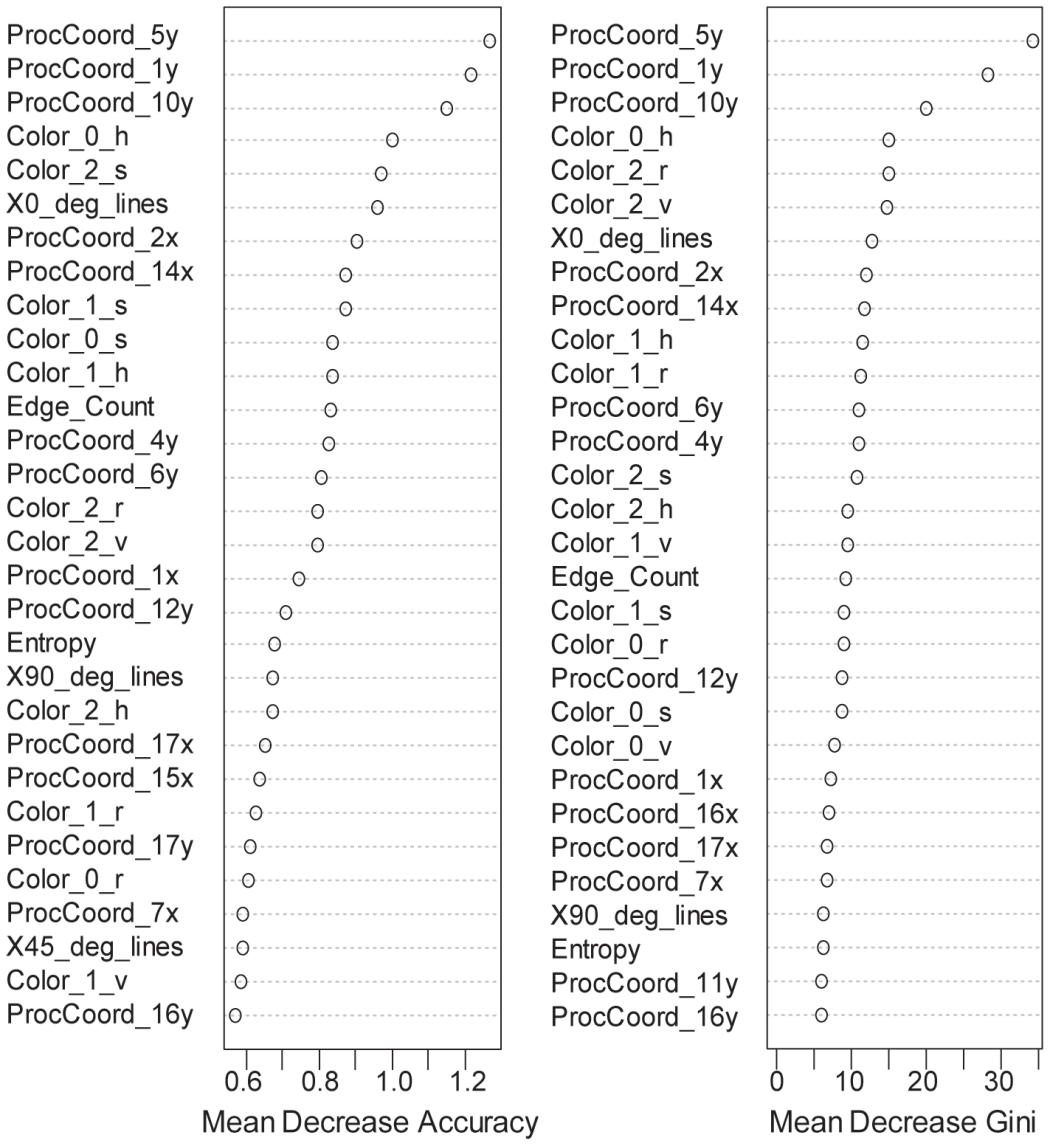
The important variables for random forests (RF). Two measures of accuracy and Gini index were plotted for each variable used for classification with random forests in a rank order from top to bottom. Meaning of abbreviations of shape variables: ProcCoord_5y, Procrustes *y* coordinate of landmark position 5; ProcCoord_2x, Procrustes *x* coordinate of landmark position 2; the number represents landmark position as described in Figure S1. The other abbreviations of shape variables follow this regularity. Meaning of abbreviations of color variables: Color_0_h, hue of the most frequent color in terms of pixel number in a given image of fish; hereafter the numbers (0, 1 and 2) between underscores represent the three most frequent colors in decreasing order of proportion. The letters at the end of the abbreviation came from RGB and HSV color models: r, red; g, green; b, blue; h, hue; s, saturation; v, value. The abbreviations of variables related with stripe pattern: X0_deg_lines, the number of straight horizontal lines; X90_deg_lines, the number of straight vertical lines; X45_deg_lines, the number of straight diagonal lines.

**Figure 11:**
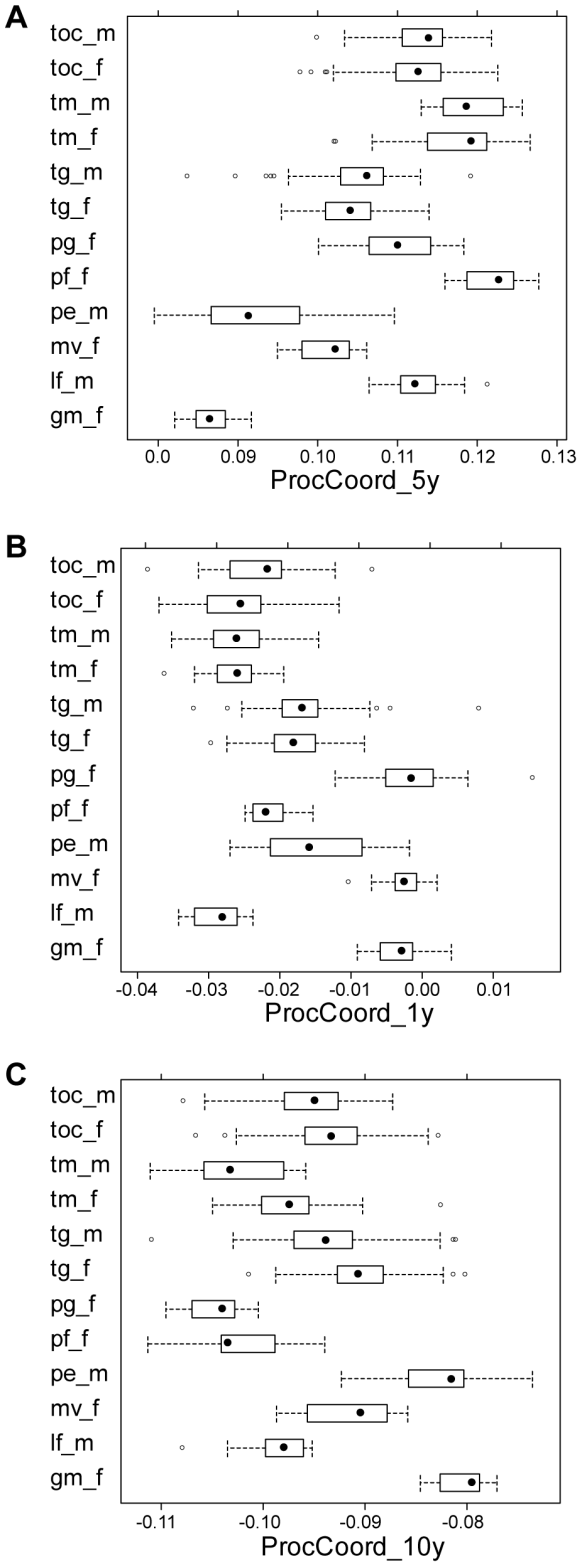
Distribution of three most important shape variables in random forests (RF) across 12 classes of cichlids. The labeling of 12 classes of cichlid fish follows the abbreviating scheme listed in Table 1 and that of shape variables of A, B and C does the previously explained one (Fig. 10).

We found that a small number of variables are very important to make an accurate RF classification. The accuracy and Gini indices revealed that shape variables came out of *y* coordinate of three landmark positions, 5, 1 and 10 in order, were the three most important variables (Fig. 10 and S1). The *y* coordinate represents a vertical height of each landmark position. Therefore the *y* coordinates of position 5 and 10 relate with the body height of cichlids and that of position 1 does with the height of mouth tip. Accordingly there were substantial differences of the distributions for these variables, as we can see from the box plots of these variables for each class (Fig. 11). The next top forth variable came from a color component, hue of the most frequent color (Fig. 10). Although the stripe pattern of cichlids did not make a meaningful contribution to the classification, the number of horizontal lines was a little bit more informative than the edge count and entropy (Fig. 10).

To investigate related factors for the success of SVM classification, we statistically examined the effect of sample size on the two kinds of estimates shown in Table 4. As expected, there was a highly significant correlation between the sample size of cichlid individuals (Table 1) and “Sum” (Table 5) (Spearman's *ρ* = 0.972, *P*<0.001). However, when we tested the correlation between the sample size and “Accuracy” (Table 5), this correlation was found to be insignificant (Spearman's *ρ* = 0.077, *P* = 0.811). Also, no correlation was found between “Sum” and “Accuracy” (Spearman's *ρ* = −0.035, *P* = 0.914). The trend of these statistics was repeated in the case of RF (data not shown). Therefore the success of SVM and RF classification did not depend on the sample sizes in this study.

### Human Survey

Our survey showed that the average success rate of accurate classification by naked human eye was 41.6%. The accuracy was not different between the two groups of students: one group questioned with original photographic images with background (average  = 41.27%; standard error  = 2.18%) and the other group questioned with grabcut images (average  = 41.96%; standard error  = 3.57%) (Table S2, Fig. 4). Both of these statistics consistently showed that untrained human ability to classify the 12 classes of cichlids was much lower than those of SVM and RF based on computer vision regardless of whether or not GM information was added. The confusion matrices of human survey revealed frequently misclassified classes of cichlids (Table S3 and S4). When both matrices were summed, the most inaccurate cases were in order of toc_f (accuracy  = 14.2%), tm_f (20.0%), toc_m (29.5%) and tm_m (32.5%). This result was very similar to that of computer vision except for the toc_f that had the most frequent misclassification to tm_f in the human survey. Also tm_f was frequently misclassified to toc_f & _m. Again it was notable that even the computer vision also made a most frequent mistake with tm_f, severely confusing it to toc_f (Table 5).

## Discussion

As the general aim of ‘automated species identification’ has been intended to reduce the burden of routine identification of vast numbers of specimens [18], we constructed an automatic pipeline that can be applied to a large set of photographic images of cichlid fishes of Lake Malawi, Africa. Admittedly much is needed to improve the degree and performance of automation. It is also needless to say that the inevitable variation of pose, illumination and ontogenic change of live fishes in wild habitats must be accounted for a working ‘automated species identification’ in the future. In the present study we rather focused on the feasibility of ‘automated species identification’ through the examination of the amount of variation extracted from diverse colors and stripes of rapidly evolving cichlids. For this purpose, we had to explore the unknown space of the feature of color and stripe to find exploitable sources of variation between both taxonomic units and sexes. This task was implemented by designing the pipeline in Figure 2 and subsequent investigations of the optimal options and parameters for each step of it. The feasibility of this pipeline was finally evaluated through its performance in species and sex identification.

To the best of our knowledge, this is the first experiment of computer vision for the analysis of coloration and stripe patterns of cichlids. Although the SVM and RF statistical classifiers failed to predict correct species in some cases (SVM: 32% when only color and stripe information was extracted but 22% when body shape information was added), the significant performance of the current method highlights its usability particularly in the analysis of large image data with a potential for flexible applications to different animal studies. Other studies of machine learning targeting color patterns of insects showed some varying proportion of misclassification [21], [22], indicating that color and its patterns, in nature, may be relatively elusive features for species identification, particularly in closely related species such as the current cichlid fishes.

We think that the considerable misclassification rates in the present study is primarily associated with biological characteristics of cichlids that originally cause difficulties in species identifications, rather than on the techniques employed here. For example, our survey with ten volunteers showed 58.4% of fish classification done by naked human eye was wrong. Simply this result indicates that the identification of species and sex of closely related cichlids is inherently very difficult to a non-expert mostly due to the similarity between them. We discuss this point in the following paragraphs.

From the results of SVM and RF classification (Table 3, 4, 5 and S1), and the lack of correlation between sample size and accuracy, we note that the more important factor for the accuracy of SVM classification is not the number of samples of the different classes in the present study, but rather the fundamental information residing in the original data. This implies that the information residing in the digital images might not be enough for computer vision to tease apart the difference between some species. From a different angle we can view this problem as the discrepancy between traits used in traditional taxonomy based on morphological characters and those that the method of computer vision extracts from photo images. The traditional classification of cichlids often uses some traits that are very subtle; thus, it is difficult for these features to be captured from photographs. For example, a slight variation in head and jaw morphology has been used to classify *Tropheops* [51]. The feature extractor for colors and the complexity of stripes inevitably overlooks such traits, which could lead to the considerable overlap of classes in the color space as shown in Figure 8. To compensate this problem, we added body shape information through GM from the same image set. Indeed the additional shape information enhanced the average accuracy in SVM and RF classifications by 9.5% from 68.1% to 77.6% and by 9.7% from 64.8% to 74.5%, respectively (Table 3).

Therefore, the oversight of the subtle but possibly important taxonomic key traits might contribute to some degree of misclassification. Accordingly, we can conclude that the accuracy of the statistical classifiers depends highly on the data set examined and the classes involved, and thus an extent of similarity in color and stripe patterns among classes. Although we cannot currently account for all the other sources of misclassification that were introduced by several steps of data transformation from the preparation of images in the field to the final digital classification of the feature vectors, we strongly consider two biological factors related with the rapid speciation of Lake Malawi cichlids: short divergence time and gene flow among species for the non-trivial proportion of misclassification (the least 22% by SVM in Table 3) unresolved to the last by the current methods. This reasoning calls researchers' attention to a need of study of genetic data corresponding to the individuals examined by CV and GM.

### Relevance to Genetics and Morphometrics

The CV and GM results directly raise a question. Do the frequent misclassifications in some species indeed correlate to high genetic similarities between them? To address this question we examined the presence of correlation through Mantel test of two pairwise matrices: the rate of misclassification of SVM (Table S5) and genetic distances among mitochondrial NADH dehydrogenase subunit 2 (ND2) gene sequences obtained from GenBank (mv_f: EF585270; pf_f: AF305301; toc: AF305301; pe: EF585272; tm: EF585258; tg: EF585260; gm: GU946223; *Petrotilapia nigra*: GQ422567; and lf: EF585259) (Table S6). It should be noted that this Mantel test is very limited because the gene sequences were not extracted from the specimens that we used in the present study. The misclassification matrix of RF (Table S7) was also examined for another Mantel test with the same genetic matrix. Despite the insufficient resolution of the ND2 genetic distance, we found a statistically significantly negative correlation between the matrices (Mantel's *r* = −0.259, *P* = 0.0067 for SVM) but only marginal significance in RF (Mantel's *r* = −0.199, *P* = 0.0850). The significantly negative correlation in SVM was also reproduced even when the GM information was not included (data not shown).

As expected, this result implies that misclassifications of SVM and RF are likely to occur between genetically close cichlid species. For example, we had an average of 19.3% of success rate in the case of tm_f, which was mainly misclassified as toc_m & _f (Table 4). This problem also took place in the human survey (Table S3 and S4). According to a previous phylogenetic study of cichlids [52], mitochondrial ND2 sequences were found to be identical among several different species of the genus *Tropheops*, including *T. microstoma* (tm here), *T. gracilior* (tg here), *T.* “orange chest” (toc here) and *T.* “broad mouth.” This result suggests a great genetic and morphologic similarity among the *Tropheops*, although similarity in this gene does not necessarily indicate similarity for genes that contribute to taxonomic key characters. In addition, interestingly from a taxonomic review of 13 species of the genus *Tropheops* [53], we could find a hint as to why tm_f &_m resulted in such a low accuracy in the SVM classification.

Goldstein [53] examined the morphological distinctions among 7 new species and 6 recognized species using Principal Component Analysis (PCA) on 24 different morphometric measurements and 12 meristic counts. When the result of PCA was presented in a polygon clustering diagram, *T. microstoma* (tm) always occurred very close to the polygons of *T. gracilior* (tg), which represented many individuals. Unfortunately, the analyses were based on only one *T. microstoma* (tm) specimen. In fact, the singular specimen did not fall into the PCA polygon clusters from other *Tropheops*, but it was so close to *T. gracilior* (tg) that *T. microstoma* (tm) could be possibly overlapped with *T. gracilior* (tg). The overlap of polygons was observed among tm, tg and toc in our GM examination too (data not shown).

The species rich haplochromine cichlids of Lake Malawi are very similar genetically, and different species share large portions of their genetic variation. This has been seen with allozymes [54]–[56], mitochondrial DNA [57], [58], microsatellite or short-tandem-repeat (STR) loci [59], nuclear DNA sequences [60], [61] and single nucleotide polymorphisms (SNPs) [8]. The extensive sharing of genetic variation among Malawi cichlids could be due to the persistence of ancestral variation [57], [58], [61] and/or an ongoing low level of gene flow [2]. Evidence of interspecies gene flow comes from hybrids and hybrid populations [62]–[64] and the phylogeny based on broad taxon sampling and usage of nuclear and mitochondrial genes [7]. Even in locations where the local population size may not be very large, microsatellite loci generally revealed high heterozygosities, with large numbers of alleles over broad size ranges [65]–[67].

On the other hand, parallel adaptation was also suggested for the color motifs that re-occur within the same species and species complexes at different locations around the lake, as well as in multiple genera [68]. Our previous population genetic and phylogenetic studies also indicated a signature of gene flow among species [69], [70]. If hybridization occurs at low levels, then this could serve both as the source of genetic variation for color patterns and as the source of shared color motifs. Newly introduced color pattern alleles would typically be selected against but might sometimes be favored depending on the cues preferred by females [71]. Kocher and colleagues have argued in support of the “divergence with gene flow” model of speciation [72], [73] for Malawi cichlids [2], [63], [74]. On the other hand selection for character displacement among sympatric species could lead to dissimilar yet closely related sympatic species and leave higher levels of similarity between unrelated species that live in different regions [68].

Genetic similarity is the basis of phenotypic similarity. Therefore, the very limited morphological traits used for taxonomic identification, such as a slight variation in head and jaw shape that is actually used as an operational classification key in the field would be difficult to be captured by the current image processing method. One of the cases is, for example, the genus *Tropheops*, which has an unique head profile and is readily distinguished from other genera [51]. Because shape characters among *Tropheops* species would not be easily discernable to human eye, we expected that an additional explicit method such as landmark-based GM techniques might enable us to gain a much higher resolving power for solving the slight differences [62], [63], [75]–[81]. This turned out to be the case in our experiment, although the degree of increment was limited. Out of 12 classes, eight showed increased accuracies in classification but four classes did very slightly decrease (Table 4 and 5).

Nevertheless the presence of mixed genotypes among closely related cichlid species and even across genera casts doubt on the presence of clear-cut species and/or population boundaries in morphological space. Thus, this problem might have naturally contributed to the some failure of classification both in SVM and RF. It should be noted that we overlooked the possibility of the presence of hybrids in the field when classifying and labeling the fish, and thus our species identification was dichotomous regardless of their possible status as of hybrids and/or backcrosses to their parental species. Such dichotomous identifications might possibly contribute to the failure of SVM and RF.

The portion of the discrepancy (22% in SVM and 25% in RF) between our methods and traditional taxonomic classification could be partially accounted for by mixed patterns of colors and strips among species due to recent and prolonged introgression among some species. In particular, the individuals that SVM most frequently failed to correctly classify might be the fishes with atypical coloration and faintly marked strips due to hybridization or introgression of genes from different species. As a working hypothesis, we can predict that the most frequently misclassified fishes might have mixed or co-segregating genotypes between species due to short divergence times and/or gene flow. The prediction, therefore, warrants further study through population genetics in parallel with the present CV and GM analyses.

In particular, it will be very interesting to compare vector data from the feature extractor with corresponding population genetic data to see if there is any correspondence between the two independent matrices. Our current feature extractor can provide us with the whole vector values in a text format file, so we will be able to treat the data as independent multivariate data. As an easy and quick way for checking the correspondence, we can design an experiment that overlays the genetic data to that of the feature vector, focusing on hybrid-like individuals screened by genetic data such as multiple microsatellite loci or SNPs; particularly, we will be able to assess if genetic intermediates (i.e., apparent *F*1 or *F*2 hybrids) will be also intermediates by the phenotypes erected in this study. A further promising point is that if there is correspondence, we can scrutinize which columns in the vector matrix (82 features) exhibit associations to any given genetic marker. One big advance of this line of study will be that the candidate traits exhibiting strong disjunctive patterns between species or sexes may be the ones that show the most evidence of character displacement and of being under divergent natural selection, potentially by being involved in mate recognition processes. Identifying such traits and quantifying them will be a great help for us to understand diverging processes of Malawi cichlids.

Finally, note that the vector columns do not necessarily correspond to real seemingly discrete shape morphs (bars, spots, bonds, and their colors) in the body of fish in a one-to-one manner; particularly, the shape column based on entropy corresponds to a composite character of the real characters, which is analogous to the complexity of amorphous objects like clouds. Therefore, refinements in targeting morphs might be an alternative component to the current approach as applied for the recognition of wing shape of butterfly [82].

## Conclusion

The present study is a pilot investigation before the development of full-fledged ‘automated species identification’ of cichlid fishes; here, we focused on the characterization of the coloration and stripe patterns of the species-rich rock-dwelling cichlids from Lake Malawi in terms of what features and how much of them could reliably deliver species specific information, a proxy of evolution, because these external features have long been a subject of sexual selection as a driving force of speciation. As expected our result showed that there are indeed some features, frequent colors, horizontal lines and edge counts, that deliver reliable information on the species and sex identification of cichlids, with a considerable limitation too. Given this limitation and that the traditional taxonomy of cichlids and other animals is centered on morphology, other phenotypic features such as body shape variables are warrantable for the improvement of automatic classification. Evidently our result supports this notion by boosting the performance of SVM and RF classifiers; the most contributing shape variables were found to be those related to the height of body and mouth tip.

Overall, the computer vision with or without body shape information of cichlids outperformed the nonexperts' ability to discriminate different species and sexes. The average success rate in the present study seems to be slightly lower than those of other automated classification studies. The non-trivial discrepancy (minimum 22%) between the present computer vision approach and the practice of traditional taxonomy done by us in the Lake Malawi could be explained by combinations of the following reasons: (*i*) the presence of taxonomic key traits that are hard to capture with the current photographic images taken from only front side, (*ii*) the extremely close genetic distances between some cichlid species due to short divergence times; and (*iii*) putatively a considerable degree of gene flow between different species which, in turn, might have caused a certain degree of mixed patterns of color and stripe among species. If the latter reason is the case, it will be difficult to achieve a perfectly operating automated species identification system whatever currently and later available tools and techniques are introduced. This notion has implications for ‘automated species identification’ in other closely related organisms. However, the situation of short divergence times and gene flow between cichlid species provides very valuable natural mating experiments from which all the combined data of computer vision, GM and population genetics could be extracted and used to find genetic loci responsible for evolutionarily important phenotypes of color, stripe and body shape of the rapidly speciating species.

## Supporting Information

Figure S1 **Landmarks of geometric morphometric (GM) analysis of cichlids.** The numbering and dots represent 17 landmark positions for capturing body shape of cichlids.
(TIF)

Table S1 **Confusion matrix of RF on coloration and stripe vectors with GM information.**
(DOCX)

Table S2 **Summary of survey result.**
(DOCX)

Table S3 **Confusion matrix of human survey on the images with background.**
(DOCX)

Table S4 **Confusion matrix of human survey on the images without background by grabcut.**
(DOCX)

Table S5 **Matrix of pairwise misclassification rates of SVM on the combined vectors of coloration, stripe and GM information.**
(DOCX)

Table S6 **Pairwise genetic distances (** ***K2P*** **) of mitochondrial NADH dehydrogenase subunit 2 (ND2) gene sequences.**
(DOCX)

Table S7 **Matrix of pairwise misclassification rates of RF on the combined vectors of coloration, stripe and GM information.**
(DOCX)
