# Supplementary figures and images for: Identification of Cichlid Fishes from Lake Malawi Using Computer Vision
Source: PLoS One. 2013 Oct 25;8(10):e77686. doi: 10.1371/journal.pone.0077686 (PMC3808401; doi:10.1371/journal.pone.0077686)

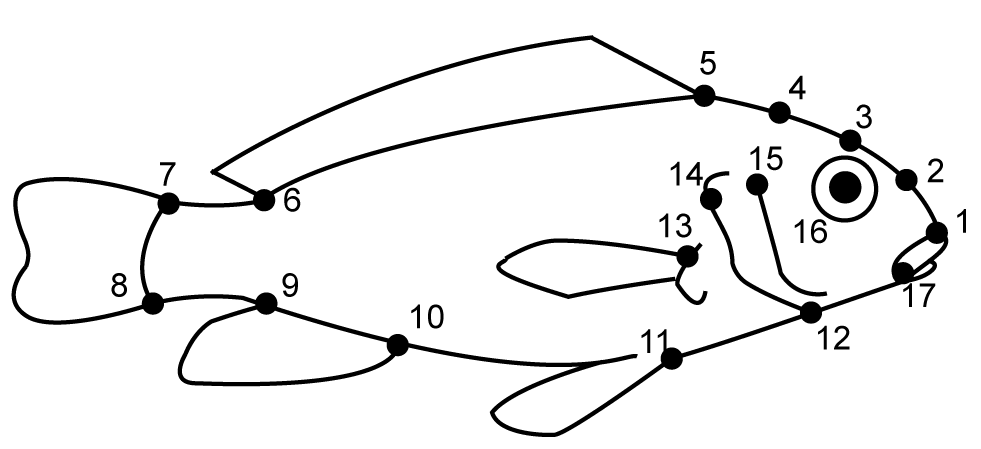

Supplement: Figure S1 — Landmarks of geometric morphometric (GM) analysis of cichlids. The numbering and dots represent 17 landmark positions for capturing body shape of cichlids. (TIF) [file pone.0077686.s001.tif]
