# Supplementary material for: Identification of Cichlid Fishes from Lake Malawi Using Computer Vision
Source: PLoS One. 2013 Oct 25;8(10):e77686. doi: 10.1371/journal.pone.0077686 (PMC3808401; doi:10.1371/journal.pone.0077686)
Supplement: Table S1 — Confusion matrix of RF on coloration and stripe vectors with GM information. (DOCX) [file pone.0077686.s002.docx]

**Table S1:** **Confusion matrix of RF on coloration and stripe vectors with GM information**

| **Predicted** | **Actual** | | | | | | | | | | | |
| --- | --- | --- | --- | --- | --- | --- | --- | --- | --- | --- | --- | --- |
|  | gm_f | lf_m | mv_f | pe_m | pf_f | pg_f | tg_f | tg_m | tm_f | tm_m | toc_f | toc_m |
| gm_f | 130 | 0 | 0 | 8 | 0 | 0 | 0 | 22 | 0 | 0 | 0 | 0 |
| lf_m | 0 | 241 | 0 | 0 | 0 | 0 | 0 | 0 | 0 | 12 | 0 | 0 |
| mv_f | 14 | 0 | 127 | 0 | 0 | 0 | 0 | 1 | 0 | 0 | 0 | 0 |
| pe_m | 71 | 0 | 0 | 473 | 0 | 0 | 0 | 46 | 0 | 0 | 11 | 20 |
| pf_f | 0 | 0 | 0 | 0 | 203 | 0 | 0 | 0 | 0 | 0 | 20 | 0 |
| pg_f | 0 | 0 | 15 | 0 | 8 | 478 | 0 | 0 | 0 | 0 | 1 | 4 |
| tg_f | 2 | 0 | 48 | 23 | 0 | 4 | 3483 | 292 | 37 | 0 | 315 | 18 |
| tg_m | 1 | 0 | 4 | 101 | 0 | 33 | 163 | 1287 | 15 | 14 | 146 | 106 |
| tm_f | 0 | 0 | 0 | 0 | 0 | 0 | 0 | 0 | 7 | 11 | 0 | 0 |
| tm_m | 0 | 2 | 0 | 0 | 0 | 0 | 0 | 0 | 33 | 135 | 0 | 0 |
| toc_f | 0 | 1 | 7 | 48 | 34 | 2 | 115 | 102 | 258 | 54 | 1615 | 444 |
| toc_m | 0 | 0 | 0 | 0 | 0 | 8 | 0 | 80 | 36 | 49 | 111 | 751 |
| Sum | 218 | 244 | 201 | 653 | 245 | 525 | 3761 | 1830 | 386 | 275 | 2219 | 1343 |
| Accuracy(%) | 59.63 | 98.77 | 63.18 | 72.43 | 82.86 | 91.05 | 92.61 | 70.33 | 1.81 | 49.09 | 55.92 | 55.92 |

From 100 repetitions of randomized experiments, 11,900 classifications were made. An average accuracy of the result is 75.04%.
