# Supplementary material for: Identification of Cichlid Fishes from Lake Malawi Using Computer Vision
Source: PLoS One. 2013 Oct 25;8(10):e77686. doi: 10.1371/journal.pone.0077686 (PMC3808401; doi:10.1371/journal.pone.0077686)
Supplement: Table S2 — Summary of survey result. (DOCX) [file pone.0077686.s003.docx]

| **Table S2:** **Summary of survey result**   \| **Image set** \| **Individual** \| **Accuracy (%)** \| \| --- \| --- \| --- \| \| **With background** \| A \| 35.26 \| \|  \| B \| 39.88 \| \|  \| C \| 38.73 \| \|  \| D \| 45.66 \| \|  \| E \| 46.82 \| \|  \| Average \| 41.27 \| \|  \| Standard error \| 2.18 \| \| **Without background** \| A’ \| 46.24 \| \|  \| B’ \| 41.61 \| \|  \| C’ \| 28.32 \| \|  \| D’ \| 47.97 \| \|  \| E’ \| 45.66 \| \|  \| Average \| 41.96 \| \|  \| Standard error \| 3.57 \| |
| --- | --- | --- | --- | --- | --- | --- | --- | --- | --- | --- | --- | --- | --- | --- | --- | --- | --- | --- | --- | --- | --- | --- | --- | --- | --- | --- | --- | --- | --- | --- | --- | --- | --- | --- | --- | --- | --- | --- | --- | --- | --- | --- | --- | --- | --- |
