# Supplementary material for: Identification of Cichlid Fishes from Lake Malawi Using Computer Vision
Source: PLoS One. 2013 Oct 25;8(10):e77686. doi: 10.1371/journal.pone.0077686 (PMC3808401; doi:10.1371/journal.pone.0077686)
Supplement: Table S3 — Confusion matrix of human survey on the images with background. (DOCX) [file pone.0077686.s004.docx]

**Table S3:** **Confusion matrix of human survey on the images with background**

| **Predicted** | **Actual** | | | | | | | | | | | |
| --- | --- | --- | --- | --- | --- | --- | --- | --- | --- | --- | --- | --- |
|  | gm_f | lf_m | mv_f | pe_m | pf_f | pg_f | tg_f | tg_m | tm_f | tm_m | toc_f | toc_m |
| gm_f | 5 | 0 | 0 | 21 | 0 | 0 | 2 | 4 | 0 | 0 | 3 | 3 |
| lf_m | 0 | 15 | 0 | 0 | 0 | 0 | 1 | 12 | 0 | 2 | 0 | 0 |
| mv_f | 0 | 0 | 14 | 0 | 6 | 15 | 1 | 0 | 0 | 0 | 0 | 0 |
| pe_m | 7 | 0 | 0 | 23 | 0 | 1 | 2 | 7 | 2 | 4 | 8 | 4 |
| pf_f | 0 | 0 | 0 | 0 | 4 | 2 | 10 | 0 | 1 | 0 | 21 | 1 |
| pg_f | 0 | 0 | 0 | 0 | 1 | 9 | 15 | 5 | 3 | 0 | 19 | 1 |
| tg_f | 0 | 0 | 0 | 1 | 1 | 0 | 178 | 14 | 1 | 0 | 4 | 8 |
| tg_m | 0 | 0 | 0 | 0 | 0 | 2 | 26 | 42 | 0 | 1 | 11 | 17 |
| tm_f | 2 | 0 | 1 | 0 | 2 | 1 | 26 | 9 | 9 | 2 | 59 | 7 |
| tm_m | 0 | 0 | 0 | 0 | 0 | 0 | 4 | 23 | 3 | 6 | 5 | 17 |
| toc_f | 1 | 0 | 0 | 0 | 1 | 4 | 9 | 5 | 5 | 1 | 27 | 11 |
| toc_m | 0 | 0 | 0 | 0 | 0 | 1 | 6 | 14 | 6 | 4 | 8 | 26 |
| Sum | 15 | 15 | 15 | 45 | 15 | 35 | 280 | 135 | 30 | 20 | 165 | 95 |
| Accuracy(%) | 33.33 | 100 | 93.33 | 51.11 | 26.67 | 25.71 | 63.57 | 31.11 | 30.00 | 30.00 | 16.36 | 27.37 |
