# Supplementary material for: Identification of Cichlid Fishes from Lake Malawi Using Computer Vision
Source: PLoS One. 2013 Oct 25;8(10):e77686. doi: 10.1371/journal.pone.0077686 (PMC3808401; doi:10.1371/journal.pone.0077686)
Supplement: Table S4 — Confusion matrix of human survey on the images without background by grabcut. (DOCX) [file pone.0077686.s005.docx]

**Table S4:** **Confusion matrix of human survey on the images without background by grabcut**

| **Predicted** | **Actual** | | | | | | | | | | | |
| --- | --- | --- | --- | --- | --- | --- | --- | --- | --- | --- | --- | --- |
|  | gm_f | lf_m | mv_f | pe_m | pf_f | pg_f | tg_f | tg_m | tm_f | tm_m | toc_f | toc_m |
| gm_f | 6 | 0 | 0 | 5 | 0 | 1 | 13 | 2 | 1 | 0 | 15 | 2 |
| lf_m | 0 | 11 | 0 | 1 | 0 | 0 | 1 | 6 | 0 | 4 | 2 | 0 |
| mv_f | 0 | 0 | 14 | 0 | 1 | 16 | 1 | 0 | 1 | 0 | 2 | 2 |
| pe_m | 2 | 1 | 0 | 19 | 0 | 0 | 1 | 3 | 0 | 2 | 8 | 6 |
| pf_f | 0 | 0 | 0 | 0 | 14 | 2 | 8 | 0 | 2 | 0 | 8 | 0 |
| pg_f | 1 | 0 | 0 | 1 | 0 | 15 | 22 | 0 | 1 | 0 | 6 | 0 |
| tg_f | 1 | 0 | 0 | 0 | 0 | 1 | 161 | 8 | 1 | 0 | 24 | 2 |
| tg_m | 0 | 0 | 1 | 5 | 0 | 0 | 16 | 65 | 1 | 4 | 10 | 32 |
| tm_f | 3 | 0 | 0 | 4 | 0 | 0 | 16 | 10 | 3 | 1 | 43 | 9 |
| tm_m | 1 | 2 | 0 | 0 | 0 | 0 | 0 | 16 | 0 | 7 | 3 | 6 |
| toc_f | 0 | 0 | 0 | 4 | 0 | 0 | 35 | 4 | 14 | 0 | 20 | 6 |
| toc_m | 1 | 1 | 0 | 6 | 0 | 0 | 6 | 21 | 6 | 2 | 24 | 30 |
| Sum | 15 | 15 | 15 | 45 | 15 | 35 | 280 | 135 | 30 | 20 | 165 | 95 |
| Accuracy(%) | 40.00 | 73.33 | 93.33 | 42.22 | 93.33 | 42.86 | 57.5 | 48.15 | 10.00 | 35.00 | 12.12 | 31.58 |
