# Supplementary material for: Identification of Cichlid Fishes from Lake Malawi Using Computer Vision
Source: PLoS One. 2013 Oct 25;8(10):e77686. doi: 10.1371/journal.pone.0077686 (PMC3808401; doi:10.1371/journal.pone.0077686)
Supplement: Table S5 — Matrix of pairwise misclassification rates of SVM on the combined vectors of coloration, stripe and GM information. (DOCX) [file pone.0077686.s006.docx]

**Table S5: Matrix of pairwise misclassification rates of SVM on the combined vectors of coloration, stripe and GM information**

| Species name | gm_f | lf_m | mv_f | pe_m | pf_f | pg_f | tg_f | tg_m | tm_f | tm_m | toc_f | toc_m |
| --- | --- | --- | --- | --- | --- | --- | --- | --- | --- | --- | --- | --- |
| gm_f |  |  |  |  |  |  |  |  |  |  |  |  |
| lf_m | 0.0000 |  |  |  |  |  |  |  |  |  |  |  |
| mv_f | 0.0356 | 0.0000 |  |  |  |  |  |  |  |  |  |  |
| pe_m | 0.1449 | 0.0548 | 0.0000 |  |  |  |  |  |  |  |  |  |
| pf_f | 0.0000 | 0.0000 | 0.0132 | 0.0000 |  |  |  |  |  |  |  |  |
| pg_f | 0.0000 | 0.0000 | 0.0000 | 0.0018 | 0.0000 |  |  |  |  |  |  |  |
| tg_f | 0.0000 | 0.0000 | 0.0654 | 0.0213 | 0.0000 | 0.0038 |  |  |  |  |  |  |
| tg_m | 0.0522 | 0.0000 | 0.0000 | 0.1042 | 0.0000 | 0.0262 | 0.0870 |  |  |  |  |  |
| tm_f | 0.0072 | 0.0408 | 0.0000 | 0.0000 | 0.0000 | 0.0000 | 0.0255 | 0.0072 |  |  |  |  |
| tm_m | 0.0000 | 0.0306 | 0.0000 | 0.0000 | 0.0000 | 0.0000 | 0.0000 | 0.0177 | 0.1440 |  |  |  |
| toc_f | 0.0000 | 0.0000 | 0.0000 | 0.0082 | 0.0023 | 0.0077 | 0.0696 | 0.0647 | 0.2212 | 0.0422 |  |  |
| toc_m | 0.0000 | 0.0064 | 0.0000 | 0.0059 | 0.0068 | 0.0095 | 0.0083 | 0.0638 | 0.1233 | 0.0560 | 0.1735 |  |

For the conversion of the rectangular confusion matrix (Table 5) to this triangular misclassification matrix, we used an average value of two misclassification rates as a single estimate of a pairwise comparison between two species compared; for example, the confusion rate of mv_f to pf_f (1.27%) and pf_f to mv_f (1.37%) in Table 5 was arithmetically averaged (1.32%).
