# Supplementary material for: Identification of Cichlid Fishes from Lake Malawi Using Computer Vision
Source: PLoS One. 2013 Oct 25;8(10):e77686. doi: 10.1371/journal.pone.0077686 (PMC3808401; doi:10.1371/journal.pone.0077686)
Supplement: Table S6 — Pairwise genetic distances ( K2P ) of mitochondrial NADH dehydrogenase subunit 2 (ND2) gene sequences. (DOCX) [file pone.0077686.s007.docx]

**Table S6: Pairwise genetic distances (*K2P*) of mitochondrial NADH dehydrogenase subunit 2 (ND2) gene sequences**

| Species name  (GenBank Acc. #) | gm_f | lf_m | mv_f | pe_m | pf_f | pg_f | tg_f | tg_m | tm_f | tm_m | toc_f | toc_m |
| --- | --- | --- | --- | --- | --- | --- | --- | --- | --- | --- | --- | --- |
| *Genyochromis mento*  (GU946223) |  |  |  |  |  |  |  |  |  |  |  |  |
| *Labeotropheus fuelleborni*  (EF585259) | 0.0022 |  |  |  |  |  |  |  |  |  |  |  |
| *Melanochromis vermivorus*  (EF585270) | 0.0022 | 0.0022 |  |  |  |  |  |  |  |  |  |  |
| *Pseudotropheus elongates*  (EF585272) | 0.0032 | 0.0011 | 0.0032 |  |  |  |  |  |  |  |  |  |
| *Protomelas fenestratus*  (AF305301) | 0.0274 | 0.0252 | 0.0274 | 0.0263 |  |  |  |  |  |  |  |  |
| *Petrotilapia nigra*  (GQ422567) | 0.0022 | 0.0022 | 0.0000 | 0.0032 | 0.0274 |  |  |  |  |  |  |  |
| *Tropheus gracilior*  (EF585260) | 0.0022 | 0.0022 | 0.0000 | 0.0032 | 0.0274 | 0.0000 |  |  |  |  |  |  |
| *Tropheus gracilior*  (EF585260) | 0.0022 | 0.0022 | 0.0000 | 0.0032 | 0.0274 | 0.0000 | 0.0000 |  |  |  |  |  |
| *Tropheus microstoma*  (EF585258) | 0.0022 | 0.0022 | 0.0000 | 0.0032 | 0.0274 | 0.0000 | 0.0000 | 0.0000 |  |  |  |  |
| *Tropheus microstoma*  (EF585258) | 0.0022 | 0.0022 | 0.0000 | 0.0032 | 0.0274 | 0.0000 | 0.0000 | 0.0000 | 0.0000 |  |  |  |
| *Tropheops* sp. "orange chest"  (GQ422583) | 0.0022 | 0.0022 | 0.0000 | 0.0032 | 0.0274 | 0.0000 | 0.0000 | 0.0000 | 0.0000 | 0.0000 |  |  |
| *Tropheops* sp. "orange chest"  (GQ422583) | 0.0022 | 0.0022 | 0.0000 | 0.0032 | 0.0274 | 0.0000 | 0.0000 | 0.0000 | 0.0000 | 0.0000 | 0.0000 |  |

The ND2 sequence of *Petrotilapia nigra* was used for the congeneric species *Petrotilapia genalutea* (pg) due to the absence of a sequence for pg. Additionally, because the classification by SVM and RF treated “male” and “female” of the same species separately, we conservatively used the same specific ND2 sequence for both “male” and “female” twice for the species with both sexes.
