# Supplementary material for: Identification of Cichlid Fishes from Lake Malawi Using Computer Vision
Source: PLoS One. 2013 Oct 25;8(10):e77686. doi: 10.1371/journal.pone.0077686 (PMC3808401; doi:10.1371/journal.pone.0077686)
Supplement: Table S7 — Matrix of pairwise misclassification rates of RF on the combined vectors of coloration, stripe and GM information. (DOCX) [file pone.0077686.s008.docx]

**Table S7:** **Matrix of pairwise misclassification rates of RF on the combined vectors of coloration, stripe and GM information**

| Species name | gm_f | lf_m | mv_f | pe_m | pf_f | pg_f | tg_f | tg_m | tm_f | tm_m | toc_f | toc_m |
| --- | --- | --- | --- | --- | --- | --- | --- | --- | --- | --- | --- | --- |
| gm_f |  |  |  |  |  |  |  |  |  |  |  |  |
| lf_m | 0.0000 |  |  |  |  |  |  |  |  |  |  |  |
| mv_f | 0.0003 | 0.0000 |  |  |  |  |  |  |  |  |  |  |
| pe_m | 0.1690 | 0.0000 | 0.0000 |  |  |  |  |  |  |  |  |  |
| pf_f | 0.0000 | 0.0000 | 0.0000 | 0.0000 |  |  |  |  |  |  |  |  |
| pg_f | 0.0000 | 0.0000 | 0.0694 | 0.0000 | 0.0163 |  |  |  |  |  |  |  |
| tg_f | 0.0046 | 0.0000 | 0.1194 | 0.0176 | 0.0000 | 0.0038 |  |  |  |  |  |  |
| tg_m | 0.0083 | 0.0000 | 0.0100 | 0.0899 | 0.0000 | 0.0314 | 0.1015 |  |  |  |  |  |
| tm_f | 0.0000 | 0.0000 | 0.0000 | 0.0000 | 0.0000 | 0.0000 | 0.0479 | 0.0194 |  |  |  |  |
| tm_m | 0.0000 | 0.0259 | 0.0000 | 0.0000 | 0.0000 | 0.0000 | 0.0000 | 0.0255 | 0.0627 |  |  |  |
| toc_f | 0.0000 | 0.0020 | 0.0174 | 0.0392 | 0.0739 | 0.0021 | 0.0863 | 0.0608 | 0.3342 | 0.0982 |  |  |
| toc_m | 0.0000 | 0.0000 | 0.0000 | 0.0074 | 0.0000 | 0.0091 | 0.0067 | 0.0613 | 0.0466 | 0.0891 | 0.1903 |  |
